# Cementless total hip arthroplasty for treatment of acetabular protrusion secondary to rheumatoid arthritis

**DOI:** 10.1186/s13018-023-03764-y

**Published:** 2023-04-07

**Authors:** Peng Liu, Yong-jie Qiao, Jin-peng Lou, Guoding Cao, Yanfeng Chang, Sheng-hu Zhou

**Affiliations:** 1 Department of Joint Surgery, The 940Th Hospital of Joint Logistic Support Force of Chinese People’s Liberation Army, South Binhe Road, No. 333, Lanzhou City, 730050 Gansu Province People’s Republic of China; 2 Department of Orthopaedics, Lanzhou University Second Hospital, Cuiying Gate, No. 82, Chengguan District, Lanzhou City, 730030 Gansu Province People’s Republic of China

**Keywords:** Total hip arthroplasty, Rheumatoid arthritis, Acetabular protrusion, Bone grafting, Cementless prosthesis

## Abstract

**Background:**

To explore the surgical technique and clinical outcomes of cementless total hip arthroplasty (THA) combined with impacted bone grafting for the treatment of moderate and severe acetabular protrusion with rheumatoid arthritis (RA).

**Methods:**

From January 2010 to October 2020, 45 patients (56 hips), including 17 men (22 hips) and 28 women (34 hips) with acetabular impingement secondary to RA, were treated with bioprosthetic THA combined with autologous bone grafting at our hospital. According to the Sotello-Garza and Charnley classification criteria, there were 40 cases (49 hips) of type II (protrusio acetabuli 6–15 mm) and 5 cases (7 hips) of type III (protrusio acetabuli > 15 mm). At the postoperative follow-up, the ROM of the hip joint, the VAS score, and the Harris score were evaluated. The healing of the bone graft, the restoration of the hip rotation center, and the prosthesis loosening were assessed by plain anteroposterior radiographs.

**Results:**

The average operation time was 95.53 ± 22.45 min, and the mean blood loss was 156.16 ± 69.25 mL. There were no neurovascular complications during the operation. The mean follow-up duration was 5.20 ± 1.20 years. The horizontal distance of the hip rotation center increased from preoperative 10.40 ± 2.50 mm to postoperative 24.03 ± 1.77 mm, and the vertical distance increased from preoperative 72.36 ± 3.10 mm to postoperative 92.48 ± 5.31 mm. The range of flexion motion of the hip joint increased from 39.48 ± 8.36° preoperatively to 103.07 ± 7.64° postoperatively, and the range of abduction motion increased from 10.86 ± 4.34° preoperatively to 36.75 ± 3.99° postoperatively. At the last follow-up, the Harris score increased from 37.84 ± 4.74 to 89.55 ± 4.05. All patients were able to move independently without assistance.

**Conclusions:**

Cementless THA combined with impacted grafting granule bone of the autogenous femoral head and biological acetabular cup can reconstruct the acetabulum, restore the rotation center of the hip joint, and achieve good medium-term outcomes in the treatment of moderate to severe acetabular herniation secondary to RA.

## Background

Rheumatoid arthritis (RA) is a systemic autoimmune disease characterized by chronic and aggressive polyarticular synovitis [1]. In contrast to secondary osteoarthrosis, the femoral head is collapsed or even disappears, and the acetabulum has an irregular oval shape, and in many cases of RA, the acetabulum protrudes, with an incidence of approximately 5% [2]. Acetabular invagination can increase the weight-bearing of the acetabular bottom and further aggravate the progress of acetabular retraction. Hip osteoarthritis can elevate the hip center, resulting in shortening of the affected limb and ultimately to pain, deformity, and dysfunction [3]. The acetabular protrusion secondary to RA often results in a weak acetabular rim, a thin acetabular wall, and severe local osteoporosis. These factors significantly increased the complexity of the procedure, negatively impacted the initial stability of the cementless modular cup, and markedly increased the rate of postoperative prosthetic loosening and revision. However, how to reconstruct the hip joint and the bone grafting technique has always been a challenge for joint surgeons. The use of an auto-bone graft from the pelvic wing creates additional surgical sites that may cause postoperative pain or infection. For the use of allografts, there remains a latent risk of infection with unidentified microorganisms. To avoid the use of allografts and artificial bone, sufficient auto-bone grafting with the resected femoral head can restore the anatomical position of the acetabular rotation center, which can provide good initial stability for acetabular cup fixation, long-term stability and biological fixation [4, 5]. Additionally, it also avoids the limitations or expected adverse effects of other methods of bone grafting.

We performed total hip arthroplasty using the noncement impaction auto-bone-grafting method with the resected femoral head for moderate and severe acetabular protrusion secondary to RA. In this article, we report the detailed surgical technique and good medium-term outcomes of our cases.

## Materials and methods

### Inclusion and exclusion criteria

The inclusion criteria were as follows: (1) patients diagnosed with RA complicated by the hip joint according to the diagnostic criteria of the 2021 American College of Rheumatology Guideline for the Treatment of Rheumatoid Arthritis [6]; (2) moderate and severe in patients with acetabular protrusions in which the bottom of the acetabulum exceeded Kohler's line in the pelvic anteroposterior radiography; (3) patients who underwent THA in our hospital with the availability of complete medical records and follow-up information; and (4) all patients had informed consent to the surgery, and the study was carried out with the approval of the ethics committee. The exclusion criteria were as follows: (1) patients with acetabular protrusions caused by trauma, infection, metabolic diseases, or another cause; (2) patients with RA and any other diseases that make surgery impossible; and (3) patients with acetabular protrusions without bone grafting.

### General data

We conducted a retrospective study to analyze the clinical information of acetabular protrusion with RA treated with cementless THA combined with impacted bone grafting in our institution from January 2010 to October 2020. There were 45 patients (56 hips) with protrusion acetabula secondary to RA, including 17 cases (22 hips) of males (37.78%) and 28 cases (34 hips) of females (62.22%). Twenty patients had left lesions, 14 patients had right lesions, and 11 patients had bilateral lesions, accounting for 44.44%, 31.11% and 24.45%, respectively. The patients’ ages ranged from 45 to 68 years (mean 55.64 ± 5.38 years), with an average course of 2–16 years (average 6.55 ± 3.12 years). The main clinical manifestations were hip pain and discomfort during walking, accompanied by obvious hip motion disorder. There were 39 patients (48 hips), accounting for 86.67%, with severe abduction limitation (abduction angle ≤ 25°), and six patients (8 hips), accounting for 13.33%, with mild abduction limitation (abduction angle > 25°). In addition, four patients (4 hips) exhibited grade III muscle strength, 23 patients (29 hips) exhibited grade IV muscle strength, and 18 patients (23 hips) exhibited grade V muscle strength according to MRC muscle grading, accounting for 8.89%, 51.11% and 40%, respectively. (Table 1).

**Table 1:** Patient demographics

Demographics	Date
Patients/hips	45 (56)
Mean age	45–68 years (average 55.64 ± 5.38 years)
Course of disease	2–16 years (average 6.55 ± 3.12 years)
Side	
Left hip	20
Right hip	14
Bilateral hips	11
Gender	
Male	17 Cases (22 hips)
Female	28 Cases (34 hips)
Abductor muscle strength classification	
Grade V	18 Cases (23 hips)
Grade IV	23 Cases (29 hips)
Grade III	4 Cases (4 hips)
Classification of acetabular protrusion	
Type II	39 Cases (48 hips)
Type III	6 Cases (8 hips)

### Preoperative imaging

**Fig. 1:**
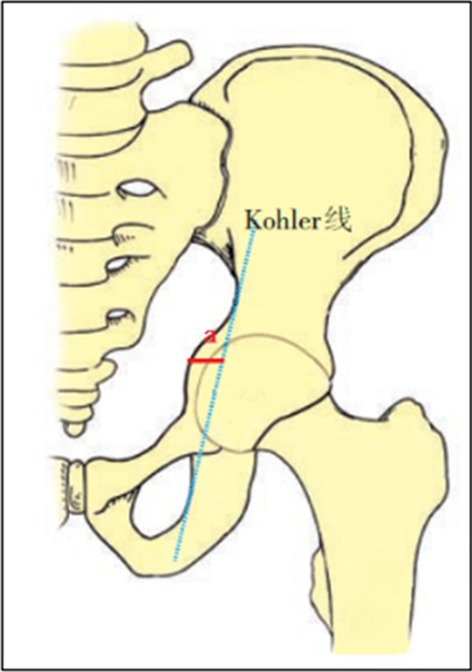
A sketch diagram of the hip joint shows how the distance of pelvic invagination distances was calculated. Kohler's line (or Nelaton’s line): taking a line drawn tangentially to the bottom of between the inner edges of ischium and ilium as the reference line, the distance of acetabular protrusion is acetabular site moved over Kohler’s line (a)

Routine hip x-ray and computed tomography (CT) scans were performed preoperatively to evaluate the acetabular invagination and femoral marrow cavity. Kohler’s line was used as an anatomical reference. Acetabular invagination could be diagnosed according to the analysis of the relative positions between the acetabular wall and Kohler’s line on the plain anteroposterior (AP) radiographs (Fig. 1). According to the Sotello-Garza and Charnley classification criteria [7], 39 patients (48 hips) were classified as type II (protrusion acetabula (6–15 mm), and six patients (8 hips) were classified as type III (protrusion acetabula > 15 mm).

### Surgical technique

All patients were operated on by the same group of surgeons and performed by the chief surgeon. After combined spinal-epidural anesthesia or general anesthesia, all patients were placed contralaterally, using a posterior lateral approach. A straight skin incision of approximately 10–15 cm in length was formed starting 1 cm posterior to the tip of the greater trochanter. The gluteus medius was retracted without removal by blunt dissection, and the short extrarotators and posterior joint capsule were removed to expose the hip joint. Because the femoral head protruded intrapelvically, it was difficult to dislocate the femoral head with conventional surgical methods. In patients with moderate acetabular protrusion, the femoral neck could undergo transverse osteotomy at 1–1.5 cm above the lesser trochanter. In those with severe protrusions, the femoral head and neck were completely trapped in the acetabulum so that the femoral neck could not be performed using common osteotomy. First, the lateral part of the edge of the femoral head and neck was removed using a drill or narrow bone knife. Then, the femoral neck was partially exposed and resected. If the femoral head had retracted and adhered inside the protrusion into the acetabulum, the femoral head was cut open, and the fragments created by breakage were removed.

Acetabular preparation was performed in two stages [8]. First, in preparation of the medial floor, the acetabular file was only used to polish the cartilage until bleeding subchondral bone to avoid piercing the bottom of the acetabulum. If the medial wall is prone to thinning and the bone is severely osteoporotic, acetabular files should be avoided if possible. Instead, the curette could be used to dispose of the acetabular bottom, and wide blood oozing from the surface of the bone bed was appropriate with drilling multiple small holes on the acetabular wall using a Kirschner wire as long as the subchondral bone appears to be very sclerotic. Second, in constructing the acetabular ring, we did not start with the smallest reamers with the aim of preparing the floor first, which is different from the osteoarthritis commonly seen in standard reaming. Although a larger reamer was used to retain the subchondral bone to a size one to two sizes smaller than the final cup size for templating. The acetabulum was ground into a hemispherical shape to achieve more stable fixation between the peripheries of the cup and as wide as a band of the acetabular rim as possible. With regard to the preparation methods of grafting bone, the resected femoral head was trimmed into irregular granules (0.5–1.0 cm), and the bottom of the acetabulum was filled with an acetabular file. A trial head with a size smaller than the diameter of the acetabulum was used for the impaction of the grafting bone until rigid compression. The acetabular prosthesis should be in contact with at least 60% to 70% of acetabular bone for adequate stability of the cup to facilitate one ingrowth into the shell and provide sufficient mechanical support to the underlying graft [2]. Finally, the acetabular prosthesis of the same size was placed at 45° abduction and 15°–25° anteversion angle, and the cementless acetabular component was inserted and fixed with a supplemental screw. For patients with severe acetabular wall defects, we added extra bone supplied from the pelvic wing to better reconstruct the acetabular reinforcement ring due to insufficient femoral head resection.

All the patients in this study were treated with biological total hip prostheses. A porous tantalum acetabular cup (TM Cup, Zimmer, USA) was selected in 35 patients (42 hips) with incomplete acetabular rings and poor quality of the acetabular bed. Another 10 patients (14 hips) with intact acetabular rings and sufficient bone grafting were placed in sintered, three-dimensional, asymmetric, titanium, and porous-coated cups (R3 Acetabular system, Smith & Nephew, USA). The other components of the THA that were used in this series were as follows: The weight-bearing interfaces of 20 patients (24 hips) received the fourth-generation ceramic-on-ceramic implantations, and ceramic-on-highly cross-linked polyethylene were placed in 25 patients (32 hips).

### Postoperative follow-up and evaluation

All patients were followed up regularly at 1 month, 2 months, 3 months, 6 months, 12 months, and once a year after 1 year after the operation. The operation time, intraoperative blood loss, and postoperative complications of all patients were recorded. The visual analog scale (VAS) [9] was used to evaluate hip pain before and after surgery. The Harris score system [10] was used to assess hip joint function before and after surgery. The length difference of the lower limbs was measured before and after the operation, and the length correction of the affected limbs was evaluated (the length of the lower limbs was the distance from the anterior superior iliac spine to the tip of the medial malleolus).

We assessed the recovery of the rotation center of the hip on anteroposterior radiographs of the pelvis immediately postoperatively. This was measured by (i) the horizontal distance of the new hip center to Kohler's line and (ii) the vertical distance of the new hip center to the interteardrop line [11]. The bone healing, bone resorption, and loosening of the prosthesis were compared immediately after surgery and follow-up radiographs. (1) Bone healing was tested according to the Engh fixation/stabilization standard [12]. Bone ingrowth was evaluated by observing whether continuous trabeculae bone passed through the interface between bone grafting and host bone on x-ray. (2) Bone resorption within the acetabular bone graft was determined according to the Gerber and Harris criteria [13]. It can be divided into three types. Mild reabsorption was defined as a lucency shadow around the graft of less than 33%. Severe reabsorption was defined as the lucency shadow around the graft of more than 50% of the graft. In comparison, a lucency shadow at approximately 33–50% of the graft reflected moderate reabsorption. (3) Loosening of the acetabular prosthesis was reflected by a shift (horizontal or vertical) of the acetabular cup of > 5 mm or the presence of transparent lines around the prosthesis [14].

### Statistical analysis

SPSS software (v25.0, SPSS Inc. USA) was used to analyze all the data. The measurement data were presented as the means ± standard deviations ($\overline{x}$ ± s). The measurement data of patients in the same group before and after the operation were analyzed by paired t tests, and the counting data were analyzed by *χ*^2^ tests. *P* < 0.05 was considered statistically significant.

## Results

### Operational data

The operation was completed successfully in 45 patients (56 hips). The operation time ranged from 80 to 140 min (mean 95.53 ± 22.45 min). The average intraoperative blood loss ranged from 80 to 400 mL (mean 156.16 ± 69.25 mL). The average intraoperative volume of bone graft ranged from 32 to 55 cm^3^ (mean 40.59 ± 5.53 cm^3^). No intraoperative complications such as neurovascular injury or femoral or acetabular fractures occurred. Retrograde removal of the femoral head was used in 45 patients (56 hips). Osteotomy of the femoral neck was completed in two patients (2 hips) through greater trochanter osteotomy. After prosthesis implantation, reduction of the hip joint was extremely difficult in three patients (4 hips), the femoral calcar had to be shortened, and the femoral stem moved downward.

The average depth of acetabular invagination before the operation was (10.97 ± 3.08) mm, ranging from 6.9 to 18.2 mm. According to the Sotello-Garza and Charnley classification criteria [7], 39 patients (48 hips) were type II, and six patients (8 hips) were type III. The femoral head was trimmed with irregular particles, and the bottom of the acetabulum was filled to restore the normal center of rotation as much as possible. The horizontal distance between the center of the femoral head and Kohler’s line increased from preoperative 7.40–12.70 mm (mean 10.40 ± 2.50 mm) to postoperative 21.30–28.40 mm (mean 24.03 ± 1.77 mm), and the vertical distance between the center of the femoral head and the line joining bilateral ischial tuberosities decreased from preoperative 81.10–104.50 mm (mean 72.36 ± 3.10 mm) to postoperative 67.70–80.50 mm (mean 92.48 ± 5.31 mm); the difference was statistically significant (*P* < 0.01).

### Follow-up joint function

The mean length of stay for all patients was 9.66 ± 1.83 days, ranging from 7 to 14 days, and all patients were followed up for 2 to 8 years (mean 5.20 ± 1.20 years). The range of flexion motion of the hip joint increased from 25° to 55° (mean 39.48 ± 8.36)° preoperatively to 90°–115° (mean 103.07 ± 7.64)° at the final follow-up, and the range of abduction motion increased from 5° to 20° (mean 10.86 ± 4.34)° to 30°–45° (mean 36.75 ± 3.99)° at the final follow-up, with a statistically significant difference (*P* < 0.01). The average VAS score decreased from 3 to 8 (mean 5.21 ± 1.11) preoperatively to 0 to 3 (mean 0.98 ± 0.82) postoperatively, and the length difference of both lower limbs decreased from 15–35 mm (mean 23.64 ± 5.00) mm preoperatively to 1–14.00 mm (mean 5.73 ± 3.05) mm postoperatively. The Harris score increased from 30 to 50 (mean 37.84 ± 4.74) preoperatively to 80–96, with an average of 89.55 ± 4.05 postoperatively. The difference between them was statistically significant. All the patients in the last follow-up could move independently without assistance, including up and down the stairs and putting on shoes independently. The subjective evaluation was very satisfactory. (Table 2).

**Table 2:** Follow-up datas

Follow-up index	Data
Cases (hips)	45 (56)
Operation time (min)	95.53 ± 22.45
Blood loss (ml)	156.16 ± 69.25
Bone graft volume (cm^3^)	40.59 ± 5.53
Length of stay (days)	9.66 ± 1.83
Follow-up time (years)	5.20 ± 1.20
Distance of acetabular protrusion (mm)	10.97 ± 3.08
The hip rotation center (preoperative)	
Horizontal distance	10.40 ± 2.50 mm
Vertical distance	72.36 ± 3.10 mm
The hip rotation center (postoperative)	
Horizontal distance	24.03 ± 1.77 mm
Vertical distance	92.48 ± 5.31 mm
The length difference of both lower limbs (preoperative)	23.64 ± 5.00 mm
The length difference of both lower limbs (Postoperative)	5.73 ± 3.05 mm
ROM of hip joint (°) (preoperative)	
Flexion and extension motion	39.48 ± 8.36°
Abduction motion	10.86 ± 4.34°
ROM of hip joint ((°)) (postoperative)	
Flexion and extension Motion	103.07 ± 7.64°
Abduction motion	36.75 ± 3.99°
Preoperative Harris score	37.84 ± 4.74
Postoperative Harris score	89.55 ± 4.05
Preoperative VAS score	5.21 ± 1.11
Postoperative VAS score	0.98 ± 0.82

### Radiographic findings

**Fig. 2:**
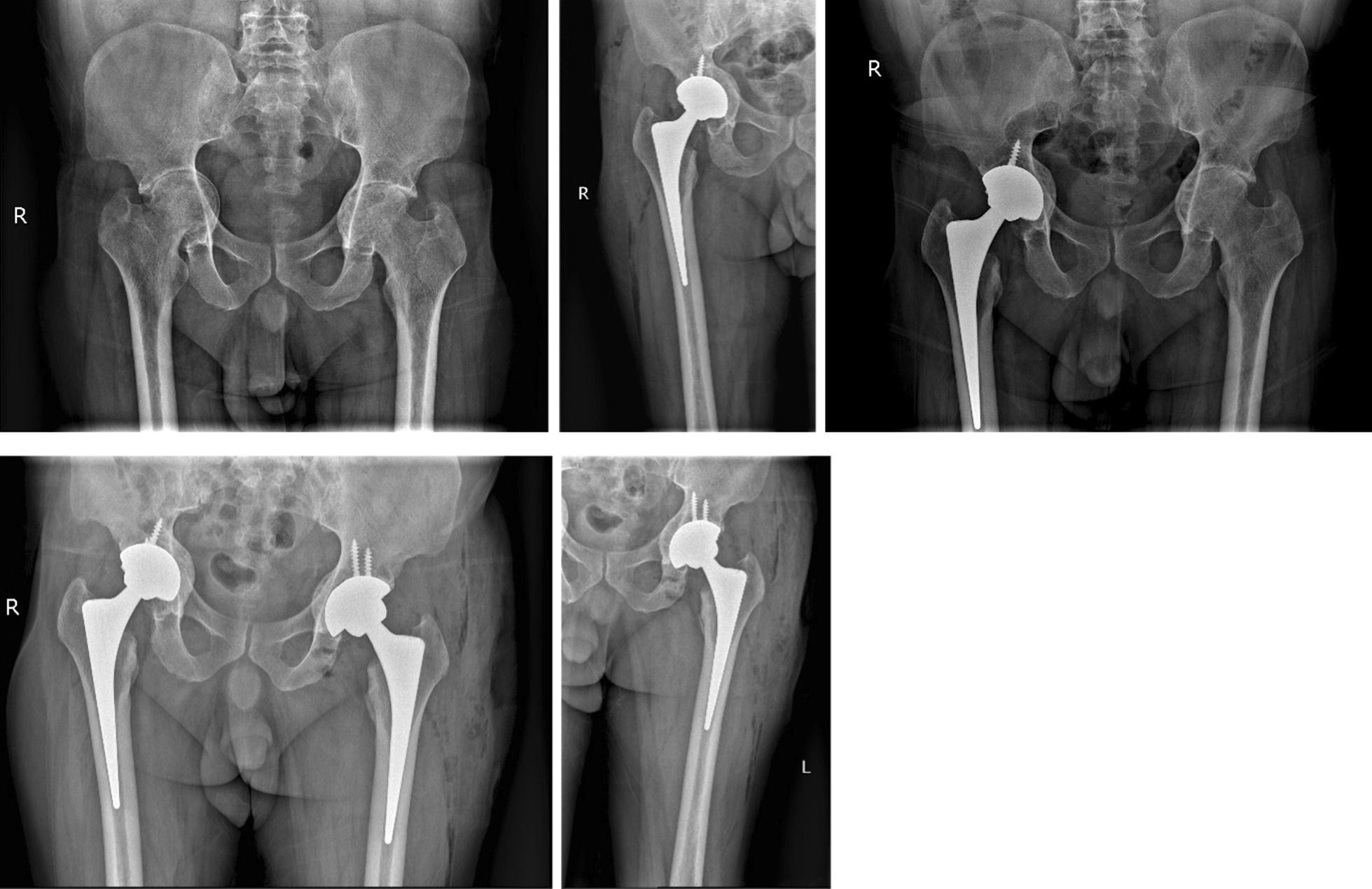
A 54-year-old female patient was diagnosed with bilateral acetabular protrusion secondary to RA. (1) Preoperative anteroposterior x-ray of the pelvis showing bilateral femoral heads protruding inward beyond the Kohler’s line. The patient was classified as type II according to Sotello-Garza and Charnley criterion. (2) Cementless THA accompanied by acetabular reconstruction using impacted bone grafting. Immediate postoperativexX-rays revealed that the acetabular prosthesis had a good position and initial stability, and the rotational center of the hip joint had returned to the normal anatomical location. (3) The 5-year follow-up after right hip replacement, there was no obvious loosening of the prosthesis, invagination of the acetabular cup, complete bone graft healing, and no bone resorption. Anteroposterior x-ray of the pelvis showed that left femoral head protruding inward beyond the Kohler’s line, which was classified as type II. (4) postoperative x-rays revealed that bilateral acetabular prosthesis had a good position and stability, and the left hip center had returned to the normal anatomical location; (5) The 2-year follow-up after left hip replacement, there was no obvious loosening of the prosthesis, and invagination of the acetabular cup

Immediate postoperative anteroposterior pelvic radiographs showed that the acetabular cup and femoral stem prosthesis were well-positioned and firmly fixed in 45 patients (56 hips). The average tilt angle of the acetabulum was 45° (range 39°–50°). In all cases, the x-ray showed that the impacted morselized bone graft was incorporated into the surrounding bone. One-year follow-up x-ray showed continued growth of the trabecular bone through the prosthetic bone interface and fusion of the graft with the host bone. At the last follow-up, the x-ray of all cases showed accurate placement of the prosthesis and good biological fixation between the prosthesis and the bone. There was no obvious transparent area between the prosthesis and bone interface, and the graft was well fused with the host bone. There was no obvious subsidence of the femoral prosthesis or loosening or invagination of the acetabular cup (Fig. 2).

## Discussion

### Clinical characteristics of acetabular protrusion secondary to RA

Acetabular invagination is a condition in which the inner wall of the acetabulum moves beyond Kohler's line, causing joint pain and disability. It can be divided into primary and secondary, with primary acetabular herniation being a rare condition whose cause is not fully understood. Otto’s disease is the most common type of primary acetabular protrusion and usually occurs in young women. Secondary acetabular protrusion is associated with a variety of diseases, which mainly include RA, ankylosing spondylitis, joint trauma, inflammatory diseases of the hip joint (such as infection), metabolic diseases (such as Paget disease), osteomalacia, rickets, Marfan syndrome, etc. [3, 4]. Among them, RA and ankylosing spondylitis are the most common. Thirty-six percent of RA patients will have clinical symptoms of hip pain combined with imaging abnormalities, and 5.2% of patients present with acetabular protrusion secondary to RA [2]. Notably, compared with acetabular invagination caused by other factors, RA is mainly characterized by synovial hyperplasia and edema of the hip joint, erosion and exfoliation of the articular cartilage, and changes in the shape of the femoral head and acetabulum [2]. Furthermore, the use of glucocorticoids in patients with RA is another important risk factor for acetabular contraction [3]. Acetabular invagination can lead to changes in the rotation center of the hip joint and the force distribution of the hip joint, which further aggravates the progress of acetabular retraction. In the force distribution of the normal hip, the femoral head receives the force from the acetabulum, which is decomposed into the force of the femoral head pointing to the acetabular floor and the force in the vertical direction. However, when acetabular invagination occurs, the force distribution of the hip joint changes again and the force of the femoral head pointing to the acetabular floor increases (Fig. 2). The aggrandize of the force will increase the weight-bearing of the acetabular bottom and further accelerate the process of acetabular invagination. The distance of acetabular retraction is approximately 2 mm every year. The retraction of the acetabulum does not cease until the invaginated greater trochanter rests against the outer edge of the acetabulum and is relatively stable [4].

Acetabular protrusion causes the hip rotation center to move upward, resulting in shortening of the affected limb, weakening gluteal muscle tension, limited flexion, abduction of the hip joint, and impingement of the hip joint [4]. THA is the most effective treatment and can relieve hip pain and restore joint function. The common manifestations of secondary acetabular invagination in RA are as follows: (1) The shape of the acetabulum is irregular shaped, ranging from a normal semicircle to oval, and may even be accompanied by an incomplete acetabular ring or bone defect at the bottom of the acetabulum. (2) The femoral head is deformed, collapsed, or even disappears. For patients with severe invagination, the femoral head and neck can be deeply trapped in the acetabulum, resulting in limited movement of the hip joint in all directions, even ankylosis. (3) Long-term wear of the acetabulum leads to thin and weak osteosclerosis at the bottom of the acetabulum and poor blood circulation on the bone surface. (4) RA patients often have varying degrees of osteoporosis, so the surrounding bone of the acetabulum is easy to compress, which accelerates the process of invagination [4]. All of the above characteristics increase the difficulty of joint replacement and the risk of THA complications, such as the initial stability of the prosthetic implant, which directly determine the success or failure of the operation.

### Surgical techniques

Patients with acetabular herniation secondary to RA usually suffer from osteoporosis, where the femoral head flattens or even disappears and the acetabular contour becomes oval with a large base and a small opening, even in combination with a disruption of the integrity of the acetabular ring or a bone defect at the base of the acetabulum. The femoral head is deeply trapped in the invaginated acetabulum, and the hip movement is significantly limited in all directions, making it difficult to dislocate, which greatly increases the difficulty of the operation. Thus, improper intraoperative dislocation can accidentally lead to acetabular wall or femur fracture [15]. In practice, the femoral head should be removed retrogradely after femoral neck osteotomy, despite the difficulty of exposing the femoral neck trapped in the acetabulum and the limited surgical space. In patients with severe invagination of the acetabulum, however, the femoral head and neck are completely sunken into the acetabulum, even ankylosis of the hip joint, so routine femoral neck osteotomy is almost impossible. Therefore, we can use a grinding drill or narrow bone knife to remove the lateral upper femoral head and upper edge of the acetabulum, with the purpose of partially exposing the protrusion of the femoral neck and allowing neck osteotomy. If the above methods still do not allow complete osteotomy of the femoral neck, the greater trochanteric osteotomy must eventually be chosen, as the following femoral neck osteotomy will be clear, safe and easy to control the direction and depth of the osteotomy. However, this technique will increase the amount of intraoperative blood loss [2]. All the patients in this group were treated with retrograde removal of the femoral head. In this study, 39 patients (48 hips) with moderate acetabular invagination could be osteotomized at one step. The other six patients (8 hips) with severe acetabular invagination needed two-step osteotomy, and two patients (2 hips) underwent femoral neck osteotomy after greater trochanter osteotomy.

The bone structure and bone strength of the acetabulum were relatively weak in RA patients. When using total hip arthroplasty for acetabular invagination, we should note that the acetabular rim may be weak, the acetabular wall may be thin, and inner acetabular defects may be present. Therefore, it is necessary to assess the outline and integrity of the acetabulum and bone defect size through preoperative radiographic images. Once a local bone defect in the acetabulum is discovered during the operation, bone grafting of the protrusio acetabuli is necessary to restore bone stock, provide a medial buttress for the cup, and adequately lateralize the cup for the restoration of the hip center. Acetabular treatment is divided into the acetabular ring and acetabular floor. First, when preparing to grind the acetabular ring, the acetabular file should be selected to be less than the acetabular cup 1–2 in size. If an acetabular file of the same size or larger is selected to grind the acetabular wall, two adverse consequences will occur. (1) Oversized acetabular file will cause further bone loss of the acetabular ring and even destroy its integrity. (2) The acetabular ring polished with the same size or larger acetabular files cannot provide hoop stress for the acetabular cup and cannot obtain early stable mechanical fixation [16]. When dealing with the thin acetabular floor, it is generally not recommended to use acetabular files that are too small to grind the thin acetabular floor so as not to aggravate the further bone loss of the acetabular floor or even penetrate the acetabular floor. In treating the hardened bone surface of the acetabular floor, we need to use a Kirschner wire to make multiple holes until the bone bed has punctate bleeding [16]. Finally, the femoral bone marrow cavity of the acetabular invagination secondary to RA is mainly characterized by thinning of the bone cortex and enlargement of the medullary cavity, which is similar to a "chimney" and is called the Dorr C femoral bone marrow cavity [17, 18]. Moreover, it is not uncommon for this type of Dorr C femoral bone marrow cavity to occur in those patients. Therefore, measurement of the femoral marrow cavity should not be overlooked in the preoperative assessment of the acetabular bone condition. After acetabular reconstruction, the rotation center of the hip joint moves downward, and the limb length is recovered, which makes joint reduction very difficult after the implantation of the prosthesis. If the postoperative limb length is more than 5 cm longer than before the operation, it is very likely to cause traction injury of the sciatic nerve [19]. It is worth noting that the integrity of the gluteus medius and iliopsoas muscles must not be disrupted; otherwise, postoperative recovery will be compromised [20].

How to handle the autologous femoral head for effective bone grafting is also a challenging technique. There are different ways to decompose the amputated femoral head, which can be divided into bone strips, granular bone, and massive bone [21]. A previous study demonstrated that cancellous bone trimmed to a volume of approximately 5 mm × 5 mm × 10 mm is suitable for bone grafting, and the granular bone implanted is compacted with the acetabular file. Meanwhile, we should avoid washing the acetabulum with normal saline to prevent the loss of osteogenic factors in the granular bone [22]. In this study, granular bone was selected for the bone graft to reconstruct the acetabulum. First, the cartilage on the surface of the amputated femoral head was removed, retaining only the cancellous bone inside, which was then decomposed into a good deal of cancellous bone block of 5–10 mm or bone fragments of approximately 5 mm thickness. Finally, the bone fragments and granular bones were implanted into the acetabulum in turn, and the bone fragments were compacted with an appropriately sized acetabular file. If there is a bone defect in the acetabular wall, the removed femoral head or autogenous iliac bone can be trimmed into a suitable curved bone for filling the bone graft. In patients with insufficient bone graft of the femoral head, allogeneic bone or autogenous iliac bone can be selected for bone grafting. Biological tantalum/titanium acetabular cups are selected to treat acetabular invagination secondary to RA because their high friction coefficient provides excellent initial stability for the prosthesis, and their high porosity is conducive to bone ingrowth, which provides good long-term biological fixation for the cup [23].

In this study, five patients (5 hips) with severe acetabular invagination had bone defects at the bottom of the acetabulum, and the removed femoral head was trimmed into a suitable curved bone for filling the bone graft.

### Medium-term follow-up outcomes

This study also revealed an increased need for blood transfusion in patients with protrusio acetabuli undergoing THA with a 28.5% incidence of transfusion in the anterior acetabulum consistent with previous literature. Lorentz et al. [24] also showed an increased need for transfusion in patients with AP, with a transfusion incidence of 24.4%. The increased complexity of these operations often requires larger surgical approaches and longer operative times. Therefore, surgeons must be aware of blood loss and be prepared for the possibility of greater transfusion. There are many unpredictable factors in this kind of patient during the operation. Therefore, it is very important to evaluate hip lesions in detail, including the morphology of the acetabulum and femoral head, the distance of invagination of the acetabulum, the bone defect of the acetabular inner wall, and the morphology of the proximal femoral medullary cavity on X-ray or CT before surgery. Only a comprehensive preoperative evaluation and personalized surgical plan can achieve good postoperative results. Therefore, when those patients undergo THA, we should consider not only the reconstruction of the acetabulum but also the soft tissue balance of the hip joint. The technical points of the operation are as follows [16]: (1) How to reshape the acetabulum and restore its integrity. (2) How can the anatomical rotation center of the hip joint be restored through sufficient bone grafting during the operation? (3) How to obtain stable compression of the acetabular cup for early stability of the acetabular cup. (4) How to release the soft tissue of contracture to restore the normal soft tissue tension around the hip joint.

The curative effect after THA in patients with acetabular protrusion secondary to RA is related to many factors, including the recovery of the rotation center, the type of prosthesis and bone graft, the amount of bone graft, and the balance of soft tissue. The use of cement acetabular prostheses to treat acetabular invagination is controversial because of the high loosening rate after cement total hip arthroplasty and the high temperature generated during cement formation, which can damage the bottom of the acetabulum, further aggravating the depth of invagination and even penetrate into the acetabular floor [25]. Allogeneic bone grafts have some disadvantages, such as rejection, poor support strength, and poor integration with host bone, whereas autologous bone grafts have significantly lower risks. The amputated femoral head used as the source of bone graft not only achieves a good effect in the treatment of acetabular protrusion but also reduces the secondary injury of autogenous ilium [26]. In addition, the size of the bone graft is also one of the factors affecting the postoperative curative effect, and the suitable size is approximately 5–10 mm. If the cancellous bone is too large, it may be difficult to perform intraoperative prosthesis installation and acetabular shaping. The bone graft, on the contrary, is too small to provide adequate support [22]. A study found that the loosening rate of the acetabular prosthesis after THA is closely related to the distance from the rotation center of the acetabular prosthesis to the movement center of the hip joint [27]. When the center of rotation of the hip joint is restored to the anatomical position, the postoperative loosening rate of the acetabular cup is approximately 8%. If the distance between the rotation center of the hip joint and the anatomical center exceeds 10 mm, the incidence of acetabular prosthesis loosening is as high as 50% [28]. If the hip rotation center is far away from the anatomical rotation center after acetabular reconstruction, it will significantly increase the risk of prosthesis loosening (50%) and revision (24%) [4]. In patients under 50 years of age who use bone cement prostheses, the failure rate is as high as 36% after 10 to 15 years of follow-up [17]. A 20-year follow-up study found that in treating acetabular invagination with cement prostheses during hip arthroplasty, the incidence of postoperative acetabular loosening is up to 22%, and the incidence of revision is 8% [23]. Compared with cement acetabular prostheses, biological prostheses have obvious advantages in patients under 50 years old. Berend et al. [29] conducted a study to compare the long-term effects of cement prostheses and biological prostheses in the treatment of acetabular invagination. It was found that biological prostheses achieved long-term stable fixation through bone growth between the bone implant and the acetabular prosthesis. Its long-term utilization rate was higher than that of bone cement prostheses. Patients with acetabular invagination secondary to RA are often young. We should choose biological prostheses as much as possible in the selection of prostheses, which can achieve long-term biological fixation and stability and reduce the probability of prosthesis loosening and revision.

All patients in this group were grafted with autologous femoral head and/or allogeneic bone to restore the normal anatomical rotation center. Concerning the length of the contralateral femoral neck, a standard-length femoral head and neck prosthesis was selected to restore the normal femoral offset and the length of the affected limb. However, patients with acetabular invagination secondary to RA often had disuse contractures of muscles and soft tissues around the hip joint. When the rotation center of the hip joint was moved down during the operation, the surrounding soft tissue and muscles became more tense, resulting in reduced difficulties. Three patients (four hips) could not reduce the hip joint directly through traction during the operation, and the reduction was not completed until shortened osteotomy through the femoral calcar. The average Harris score and VAS score of all patients at the last follow-up were significantly higher than those before the operation. The range of motion, such as flexion, extension, and abduction of the hip joint, was greatly improved.

## Conclusion

In summary, in patients with acetabular protrusion secondary to RA, there are usually related problems such as an incomplete acetabular ring or acetabular floor defect, femoral medullary cavity enlargement, soft tissue contracture, and osteoporosis. These problems bring great difficulties and challenges to accurate osteotomy, dislocation of the femoral head, and joint reduction. Therefore, careful evaluation of the acetabular and femoral morphology and bone defects prior to THA is essential. In the process of acetabular reconstruction, the anatomical rotation center of the hip joint is restored by complete bone grafting, and the integrity of the acetabular ring is repaired if necessary to provide stable fixation for the acetabular prosthesis. In this study, biological THA combined with autologous femoral head bone grafting effectively relieved pain, restored hip joint function, and achieved an excellent medium-term effect on acetabular protrusion secondary to RA.

However, the main limitations of this study were as follows: (1) The total number of cases in this study was relatively small, and the results might be biased, which still needs to be further supported by a large sample size study. (2) The postoperative follow-up time of this study was relatively short. (3) This study was a retrospective study, and the level of evidence was not high (Table 2).

## Data Availability

The data and materials are available from the medical records department of the 940th Hospital of Joint Logistic Support Force of Chinese People’s Liberation Army. The datasets used and analyzed during the current study are available from the corresponding author on reasonable request.

## Abbreviations

THA: Total hip arthroplasty
RA: Rheumatoid arthritis
